# Hypersensitivity to metals in metal-on-metal hip arthroplasty: a prospective study of one hundred and thirty five lymphocyte transformation tests

**DOI:** 10.1007/s00264-023-05992-7

**Published:** 2023-09-28

**Authors:** Dani M. Gaillard-Campbell, Thomas P. Gross

**Affiliations:** Midlands Orthopaedics & Neurosurgery, 1910 Blanding Street, Columbia, SC 29201 USA

**Keywords:** Metal allergy, Metal-on-metal, Lymphocyte transformation test, Metal hypersensitivity

## Abstract

**Background:**

Metal allergy remains a controversial topic in the orthopaedic community. It is not known if or to what degree metal sensitivity contributes to inflammatory soft tissue failures, unexplained residual pain, or clinical complications after total joint replacement with metal prostheses.

**Methods:**

We investigated the efficacy of the lymphocyte transformation test (LTT) in predicting adverse outcomes in patients after receiving a metal joint replacement. Our study cohort consists of 135 metal-on-metal hip resurfacing arthroplasty cases performed between 2013 and 2015. All study patients had an LTT preoperatively. We retrospectively analyzed clinical outcomes and failures for our cohort.

**Results:**

There was no difference in LTT reactivity between men and women. Of the 135 patients tested, 46 (34.1% of cohort) tested positive to at least one of the materials comprising their implant, and 78 patients (57.8%) had at least one reactive score to any component of the LTT. After a minimum follow-up of two years, we did not observe an allergic response to the implant in any patients. There were no failures requiring revision. We observed a 2.2% rate of moderate residual pain; no patients with residual pain tested positive for metal sensitivity. When patients with moderate-high LTT reactivity (30.4% of cohort) were compared to the remainder of the study group, there was no difference in HHS or UCLA activity score. There was no correlation between blood metal ion levels and LTT reactivity.

**Conclusion:**

We were unable to prove any predictive value of the LTT. We failed to identify hypersensitivity to metals in patients with metal-on-metal hip resurfacing arthroplasty.

## Introduction

In the last ten years, a high failure rate with metal-on-metal (MoM) bearing hip implants renewed speculation that some patients may be allergic to metallic debris released by the arthroplasty system [1]. A number of metals comprise MoM systems, and therefore, varying amounts of metallic ions are released into the body [2, 3]. Although many of these ions occur naturally in our diet [4, 5] and can be measured in our blood, unnaturally high levels of these ions can occur in the body after joint replacement with a metallic prosthesis [2]. Surface corrosion, bearing wear, and mechanically assisted crevice corrosion at trunnions are among the mechanisms for releasing metal into the body [3]. Joint replacement implants often are subject to several of these processes. The amount of debris released primarily depends on implant design, size, and positioning [6, 7].

In the last two decades, an increasing number of hip arthroplasty failures have been reported with MoM bearings and certain trunnion types [8–10] due to adverse reactions to metal debris (ARMD). There is controversy over which factor is responsible for these failures: volume/toxicity of wear debris, trunnion corrosion debris, and/or allergic response to implant material [1, 11]. The cobalt-chrome (Co-Cr) alloy used in orthopaedic implants also contains 5–7% molybdenum, < 1% iron, and < 0.75% nickel [12], which are all found in foods like grains, beans, nuts, and chocolate [13]. Some experts have recommended avoiding Co-Cr implants in patients with a history of skin sensitivity to nickel [14, 15]. Some studies have shown more reactivity with LTTs in patients with failed implants or residual unexplained pain [16, 17]. This could be interpreted as evidence that the failure was caused by a host allergic response to the device metals. Alternatively, it is possible that these reactions are simply measuring the host response to debris. Which is the affector and effector remains unclear. Still, the LTT is increasingly being used to determine if a patient is allergic to implant material [17]. If it truly measures an allergic response to implanted metals, it could be valuable in predicting certain types of adverse clinical responses and in diagnosing the need for arthroplasty revision.

We have undertaken this study to test the hypothesis that a preoperative LTT predicts adverse outcomes with metal-on-metal hip resurfacing arthroplasty, including those that might be explained by an allergic response to metal implants.

## Materials and methods

We prospectively prescribed the LTT on 135 patients undergoing an uncemented MoM hip resurfacing arthroplasty (HRA). The primary surgeon performed all resurfacings using the fully porous Biomet Magnum-ReCap™ system using the same surgical approach as described previously [18]. None of these patients had a previous arthroplasty. The blood for the LTT was drawn in the hospital on the morning of surgery for each patient. The clinicians did not see the test results until final follow-up clinical data was fully collected. Postoperative follow-up was recommended at six weeks, one year, two years, and every other year thereafter. A metal ion test (MIT) was requested from all patients at two years postoperatively. All complications, reoperations, and revisions were recorded prospectively in our database. Once all patients reached at least two years follow-up, we compared their LTT test results, clinical outcomes, and MIT results.

Demographic information (Table 1) and surgical data (Table 2) are listed. The LTT was performed by Orthopedic Analysis Lab in Chicago, IL. For each item tested, the lab reported a lymphocyte stimulation index that had one of four possible results: non-reactive (less than 2), mildly reactive (2 to 4), reactive (4–8), and highly reactive (above 8). The LTT assessed reactivity to two different alloy particles (cobalt-chrome alloy, titanium alloy), eight metal ions (cobalt, chromium, nickel, molybdenum, vanadium, aluminum, iron, zirconium), cement monomer and particles, and a known stimulant (PHA). Table 3 presents the LTT results. Table 4 summarizes clinical outcomes, and Table 5 lists postoperative complications.

**Table 1:** Demographics

Variable	Result
Date range	1/2013–7/2015
No. of cases	135
No., % deceased	0 (0.0%)
Demographics	
No., % female	31 (23.0%)
Mean follow-up (years)	3.6 ± 0.9
Age (years)	55.7 ± 10.1
BMI	29.2 ± 6.1
T-score	0.1 ± 1.3
Diagnoses	
Osteoarthritis	112 (83.0%)
Dysplasia	9 (6.7%)
Post-trauma	2 (1.5%)
Osteonecrosis	6 (4.4%)
Other	1 (0.7%)

**Table 2:** Surgical data

Variable	Result
Length of incision (in)	4.2 ± 0.5
Operation time (min)	85.1 ± 13.0
Estimated blood loss (mL)	136.0 ± 45.2
Hospital stay (days)	1.3 ± 0.6
ASA score	1.7 ± 0.6
Femoral component size (mm)	50.2 ± 3.5

**Table 3:** LTT results

Material	Nonreactive	Mild	Moderate	High
Bulk				
Cobalt-chrome alloy	123 (91.1%)	8 (5.9%)	4 (3.0%)	0 (0.0%)
Cobalt*	134 (99.3%)	1 (0.7%)	0 (0.0%)	0 (0.0%)
Chromium*	127 (94.1%)	6 (4.4%)	2 (1.5%)	0 (0.0%)
Molybdenum*	132 (97.8%)	2 (1.5%)	1 (0.7%)	0 (0.0%)
Porous coating				
Titanium alloy	109 (80.7%)	25 (18.5%)	1 (0.7%)	0 (0.0%)
Vanadium*	125 (92.6%)	8 (5.9%)	2 (1.5%)	0 (0.0%)
Aluminum*	127 (94.1%)	5 (3.7%)	3 (2.2%)	0 (0.0%)
Other				
Iron*	125 (92.6%)	8 (5.9%)	1 (0.7%)	1 (0.7%)
Nickel*	83 (61.5%)	17 (12.6%)	10 (7.4%)	25 (18.5%)
Zirconium*	121 (89%)	12 (8.9%)	1 (0.7%)	1 (0.7%)
Cement monomer	123 (91.1%)	11 (8.1%)	1 (0.7%)	0 (0.0%)

^*^Highest available concentration

**Table 4:** Clinical results

Variable	Result
Preoperative	
HHS score	59.6 ± 13.4
Postoperative	
HHS score	97.9 ± 6.2
UCLA score	7.8 ± 2.1
VAS pain: regular	0.2 ± 0.8
VAS pain: worse	1.1 ± 1.6
Radiographic data	
AIA	34.3 ± 4.5
Under RAIL (no. hips, %)	135 (100%)
Radiolucency (no. hips, %)	0 (0.0%)
Osteolysis (no. hips, %)	0 (0.0%)

**Table 5:** Complications

Type	Result (no., %)
Fracture	1 (0.7%)
Fascia failure*	1 (0.7%)
Other	1 (0.7%)
Total complications	3 (2.2%)

^*^Reoperation

We performed all statistical analyses using XLSTAT (Addinsoft, New York, NY). Paired, 2-tailed Student’s *t*-tests were carried out to find significant differences between averages. Two sample proportion Z-tests were used to compare ratios between groups. A multivariate multiple linear regression was used to find correlation between potential explanatory variables (LTT results in Table 6; biological sex in Table 7) and selected quantitative outcomes. A logistic regression was used to model potential relationships of binary outcomes (Table 8). All tests were carried out at α = 0.05.

**Table 6:** Multivariate multiple linear regression results (LTT outcome as explanatory variable)

	Cobalt levels	Chromium levels	HHS	Pain score
*R*^2^	0.027	0.035	0.014	0.021
*F*	1.708	2.247	0.900	1.299
*Pr* > *F*	*0.196*	*0.139*	*0.346*	*0.259*

**Table 7:** Multivariate multiple linear regression results (sex as explanatory variable)

	LTT result	Cobalt levels	Chromium levels	HHS	Pain score
*R*^2^	0.001	0.030	0.026	0.000	0.061
*F*	0.034	1.922	1.671	0.001	4.035
*Pr* > *F*	*0.853*	*0.171*	*0.201*	*0.976*	0.049*

**Table 8:** Logistic regression results (complication as dependent variable)

Source	DF	Chi-square (Wald)	Pr > Wald	Chi-square (LR)	*Pr* > *LR*
LTT outcome	1	0.016	0.900	0.016	*0.899*
Sex	1	0.011	0.916	0.011	*0.916*

## Results

There was no correlation between LTT and any clinical outcome measure. There was no difference in LTT reactivity between the three patients with complications and the remaining cases without complications. One of these complications led to reoperation. There were no failures requiring revision in these 135 patients. There was no difference in the mean Harris Hip score (HHS) or University of California at Los Angeles (UCLA) activity score based on LTT reactivity. All patients with moderate-high sensitivity (*n* = 41, 30.4%) to any LTT component had an average HHS of 97.5 and UCLA activity score of 7.7 at 2-year follow-up, compared to an average HHS of 97.9 and UCLA of 7.8 among all patients tested.

In addition to implant failure, unexplained pain or adverse-wear related failure (AWRF) are two problems that could be ascribed to “allergy” [11, 17]. AWRF is defined as a severe inflammatory reaction due to excess metallic wear debris with metallosis seen at the time of revision. We observed three cases (2.2%) of residual moderate unexplained pain (defined by HHS pain score of 30 or lower). No patients with unexplained pain had a positive LTT. All acetabular components were placed within the RAIL (relative acetabular inclination limit) guidelines [7, 18]; correspondingly, there were no cases of AWRF. Of the metal ion levels, 133 (98.5%) were optimal according to the DeSmet guidelines [19] (below 4 µg/L for unilateral and 5 µg/L for bilateral resurfacings); there was no correlation between suboptimal ion levels and a reactive LTT.

There were 78 (57.8%) positive LTTs. There was no difference in the rate of positive tests between women and men. Nickel was the most associated element with a positive test (38.5%), followed by titanium alloy (19.3%). Cobalt reactivity on the LTT was present in 0.7% of patients, chromium in 6%, molybdenum in 2.3%, and nickel in 38.5%. Reactivity to Co-Cr alloy bearing surface particles was present in 9% of LTTs.

## Discussion

To our knowledge, this was the first study to investigate the efficacy of the LTT in predicting adverse responses to metal implants. Some studies have suggested metal sensitivity increases risk of residual unexplained pain, mechanical loosening, and inflammatory reactions [1, 11, 20]. In our small cohort of 135 patients, we failed to demonstrate any predictive value of the LTT. We found that 57.8% of our patients had a positive LTT prior to ever receiving a joint replacement, and of these patients, there were no failures, no unexplained pain, and only one reoperation (fascia failure). Such a large ratio of patients with LTT reactivity might lead to over-diagnosis of “metal sensitivity” and unnecessary revision surgery. This high rate of positive LTT and the lack of correlation between LTT outcome and postoperative failures suggest that this test has no value in predicting MoM HRA success or in diagnosing failure due to metal allergy.

With the exception of some well-known and poorly designed implants with extremely high failure rate, other MoM HRA systems have proven higher durability, reduced failure rates, and overall greater function [6, 21]. Despite using these otherwise well-performing implants, one group reported excessive failures due to inflammatory reactions [11]. They labeled these as “pseudotumours,” the hypothesis that these were caused by metal allergy quickly advanced [22, 23]. Women were more commonly affected; the suspected caused of this was a presensitization to metal via metal jewelry, in which women wore more often than men. No evidence was provided to support this theory. Furthermore, three recent review articles on the subject could find no convincing scientific evidence that metal allergy is a cause of joint replacement failure [14, 24, 25]. We believe that in HRA, ARMD is caused by tissue irritation from excessive wear debris, or AWRF, rather than by allergy to debris. We previously demonstrated a correlation between AWRF and acetabular malposition and described a RAIL guideline protocol for minimizing/eliminating wear failures [18]. We successfully treated cases of metallosis with acetabular revision, placing the new MoM component within the RAIL [26]. After implementing RAIL in our primary HRA cases, we increased Kaplan–Meier 10-year implant survivorship from 99 to 100% (using AWRF as the end point) [18, 27].

DeSmet first hypothesized that acetabular malposition was the cause of MoM wear failures, especially in smaller HRA components that have a lower coverage arc by design [27]. Women required smaller implants than men, on average; smaller implants are more likely to fail due to component malposition. Isaac et al. confirmed the influence of cup position on excess wear in the laboratory [28]. Our RAIL studies and reports by DeSmet and Isaac provide evidence that severe inflammatory tissue reactions around MoM HRA are a result of excess wear debris generation due to edge-loading and component malpositioning, not due to patient allergy to implant materials. The current study provides additional evidence that metal allergy is unlikely to influence ARMD failures in MoM HRA, as there is 100% implant survivorship by a minimum five years postoperatively in the 78 patients with positive LTTs.

There are several limitations to this study. First, our cohort of 135 cases is relatively small; it is possible correlations not identified herein could be found with a larger sample size. Second, this study only investigates the LTT results of patients with the Magnum-ReCap MoM HRA system. In total hip replacement, ARMD failures can occur due to mechanically-assisted crevice corrosion from the trunnion [8–10]. Also, this paper does not explore the role of LTT in predicting trunnion corrosion. Next, no failures or allergic responses were identified. Therefore, we could not test for correlation between these variables and positive LTT. However, because of the high rate of positive LTTs (57.8% with any positive LTT and 30.4% with moderate-high LTT reactivity) with noted allergic response or failure, we recommend against using this to predict adverse outcome or for patient selection.

## Conclusion

To our knowledge, this is the first attempt at validating the predictive value of LTTs. We were unable to prove the hypothesis that LTT predicts metal allergy or adverse outcomes postoperatively in MoM HRA. We found no correlation between positive LTT and clinical failure or complication, no correlation between positive LTT and unexplained pain, and no significant difference in LTT reactivity between men and women. In this study of 135 cases, LTT did not correlate with blood metal ion levels. Of patients, 57.8% had a positive LTT response, with 30.4% having a moderate to high response; none of these patients exhibited an allergic response or required revision surgery. There was no significant difference in clinical scores between patients with moderate-high LTT response and patients with none or mild LTT reactivity. The most common “allergen” was nickel at 38.5%, and cement was an “allergen” in 8.9% of cases. Until a positive predictive value of the LTT is demonstrated, we recommend that the use of this test be discontinued as a method of diagnosing or predicting failure in HRA.

## Data Availability

The dataset supporting these conclusions are available upon request.

## Abbreviations

LTT: Lymphocyte transformation test
MoM: Metal-on-metal
ARMD: Adverse reactions to metal debris
Co-Cr: Cobalt-chromium
HRA: Hip resurfacing arthroplasty
MIT: Metal ion test
HHS: Harris hip score
UCLA: University of California at Los Angeles
AWRF: Adverse wear-related failure
RAIL: Relative acetabular inclination limit
